# Towards early disease modification of Parkinson’s disease: a review of lessons learned in the Alzheimer field

**DOI:** 10.1007/s00415-020-10162-5

**Published:** 2020-08-18

**Authors:** Marthe Smedinga, Sirwan K. L. Darweesh, Bastiaan R. Bloem, Bart Post, Edo Richard

**Affiliations:** 1 Department of Neurology, Radboud University Medical Center, Donders Institute for Brain, Cognition and Behaviour, Nijmegen, The Netherlands; 2 Department of Medical Ethics, Philosophy and History of Medicine, Erasmus Medical Center, Rotterdam, The Netherlands; 3 Center of Expertise for Parkinson and Movement Disorders, Nijmegen, The Netherlands; 4 Radboud University Medical Center Alzheimer Center, Nijmegen, The Netherlands

**Keywords:** Parkinson’s disease, Alzheimer’s disease, Clinical trials, Prevention, Biomarkers, Ethics

## Abstract

Parkinson’s disease (PD) research is beginning to focus on early disease modification and prevention. The therapeutic pipeline includes a growing range of pharmacological interventions that could theoretically intervene with the underlying disease process. It is hoped that applying such interventions in a very early stage of the disease pathology, before the onset of motor symptoms or during its early stages, may prevent or delay further disease progression. To identify people in this early disease stage, criteria for ‘prodromal PD’ have been proposed—describing people with one or more specific features that jointly constitute a variably increased risk of developing clinically manifest PD. Here, we aim to draw lessons from the field of Alzheimer’s research, which has followed a similar strategy over the last decade, including the expansion of the disease label to ‘prodromal’ stages. Importantly, none of the large and costly randomized-controlled trials aiming to slow down or prevent Alzheimer’s dementia by targeting the alleged disease pathology, i.e., amyloid-β aggregation, resulted in detectable clinical effects. Lack of sufficiently robust phase 2 trial results before moving to phase 3 studies, suboptimal participant selection, insensitive outcomes, a too narrow target focus, and trial design flaws contributed to this disappointing outcome. We discuss the various similarities between these Alzheimer’s and PD approaches, and review the design of prevention or early disease modification trials for both diseases including the potential for immunotherapy. Finally, we offer considerations to optimize the design of such trials in PD, benefiting from the lessons learned in Alzheimer’s prevention research.

## Introduction

Parkinson’s disease (PD) is currently incurable. By the time of diagnosis, people with PD already have substantial and irreversible neurodegenerative pathology. For this reason, much PD research has started to focus on preventing or delaying rather than curing symptoms [1]. The interventions under study are targeted on the primary pathophysiological processes of PD, such as α-synuclein aggregation, or the glucocerebrosidase (GBA) or leucine-rich repeat kinase 2 (LRRK2) pathways, which start years before clinical symptoms appear [2]. Accordingly, prevention trials are being considered that aim to recruit relatively healthy research participants, with no or only mild symptoms, in the hope of halting the pathological process and thereby delaying or preventing the onset or progression of clinical symptoms.

With the exception of rare cases, determining in advance with certainty who will develop PD is impossible. There are also no reliable ways to measure the pathophysiological processes, e.g., α-synuclein aggregation, that are believed to cause PD. For this reason, potential research participants for trials aimed at early disease modification or prevention may be identified based on algorithms that integrate an individual’s risk profile [3, 4]. This risk is calculated by summing up the risk factors a person has for PD, ranging from sex, smoking behavior, and clinical features such as hyposmia or REM sleep behavior disorder (RBD) [4], to more advanced testing, including gene mutations, biomarkers of neurodegeneration, and subthreshold parkinsonism symptoms [3]. Those with an overall high risk of developing PD are referred to as having ‘prodromal’ PD [3, 5]; terminology which implies that they are in an early stage of PD. Yet, it may well take over 20 years before people with ‘prodromal’ PD will develop symptoms of PD, and some will never develop these at all [6].

Following the same line of reasoning, the Alzheimer’s disease (AD) label has also been expanded to ‘preclinical’ and ‘prodromal’ stages in the preparation for early disease modification and prevention trials [7]. Similarly, ‘preclinical’ and, for those with mild cognitive impairment, ‘prodromal’ AD indicate an increased risk to develop AD dementia. This risk status is based on biomarkers of amyloid-β and tau [7, 8]. Allegedly, these biomarkers reflect the pathological root of AD, analogous to the presumed role of α-synuclein in PD [9]. Therefore, in AD trials, people with elevated levels of amyloid-β and tau in the brain are identified as having an early stage of AD, even if they do not have dementia. The aim of these trials is to prevent or slow down later cognitive symptoms in these individuals by lowering levels of amyloid-β and/or tau [10, 11]. So far, however, none of the trials aiming to prevent or delay AD dementia in pre- or early symptomatic persons has led to detectable clinical effects [12, 13].

Several reasons for the lack of success of AD trials have been suggested. Suboptimal trial design (e.g., inappropriate outcome measure selection; short study duration) and lack of sufficiently robust phase 2 trial results to support a subsequent phase 3 trial are commonly listed reasons. Even though the clearance of amyloid-β did not result in cognitive benefit—undermining the hypothesis that amyloid-β is directly causal for AD dementia—the expansion of the AD label to people without dementia based on biomarkers of amyloid-β is slowly gaining momentum for implementation in clinical practice [14]. This expansion of the AD label may, however, do more harm than good in the absence of an effective treatment, especially for those who will never develop dementia [15]. This demonstrates how developments in research may tacitly impact clinical practice.

Looking back at the previous two decades of developments in AD research, we want to critically appraise the recent development to expand the PD label to ‘prodromal’ stages and, more specifically, the attempt to prevent or delay PD in these disease stages by pharmacological interventions. With several immunotherapy trials planned or ongoing in early PD [16, 17], it is vital to assure that the PD field acknowledges the lessons learned in AD immunotherapy clinical trials (see Table 1). In this paper, we aim to draw parallels between PD and AD prevention, hoping to reduce the chance of encountering repeated neutral trial results as has occurred in the AD field.

**Table 1:** Lessons learned from AD research for PD early disease modification and prevention trials

	Lessons learned from AD research for PD trials
Target population	A combination of early clinical features associated with PD and (biological) risk factors could be used as recruitment criteria, because disease progression is too advanced in people who already have PD and (biological) risk factors on itself have not enough predictive value
	If the intervention targets abnormal aggregation of a putative causal protein for which an accurately measurable biomarker exists, this biomarker status should be adopted in the eligibility criteria
Disclosing at-risk or early disease status	Apply risk terminology in communication towards research participants rather than a ‘diagnosis’ of prodromal PD
	Legal safeguards are required to protect participants against privacy violation and discrimination based on risk status
	Changes in research disease criteria may tacitly impact clinical practice
Pre-trial evidence for prevention trials	Consider targeting several putative pathological processes simultaneously—thereby not relying too heavily on one potential trigger of the pathophysiological process, provided that the underlying evidence is sufficiently supported by pre-trial evidence
Recruitment strategies	Transnational recruitment registries with clinical and biomarker information may tackle current recruitment issues, but are subject to several ethical challenges
Outcome measure selection	More sensitive outcome measures may increase the chance of finding a clinical intervention effect, but can only serve as proof of concept if not directly translatable into a clinically relevant effect
	Immunotherapy may have adverse effects and requires continued scrutiny, also in phase 3 trials
Advancing to phase 3 trials	Use sub-group analyses guidelines to prevent over-interpretation of post hoc analyses in phase 2 trials that lead to misleading expectations for phase 3 trials

The manuscript does not contain clinical studies or patient data

## Considering the expansion of the PD disease label to ‘earlier stages’

### Who to recruit for early disease modification or prevention trials?

Selecting at-risk populations is paramount for early intervention and prevention trials in PD. There are several options. First, at-risk populations can be defined based on clinical features that are supported by PD biomarkers, such as presence of a RBD or olfactory loss in combination with markers of advanced pathological evolution, e.g., dopaminergic transport imaging abnormalities. Without access to more advanced risk testing, a risk prediction can also be made on clinical observations, including complaints such as tremor, constipation, or dizziness [4]. A major advantage is that models based on such clinical risk markers have relatively high accuracy for predicting which individuals may subsequently be diagnosed with clinically overt PD [18]. However, recruiting people who are close to a PD diagnosis would leave limited room for actual early disease modification or prevention possibilities due to the generally advanced stage of neuronal loss at the moment in which first symptoms occur. This concern applies even more so to trials including recently diagnosed PD patients who averagely have a substantial reduction of dopaminergic neurons in the nigrostriatal pathway by the time of diagnosis [19]. Given the advanced level of neurodegeneration in those who (almost) meet PD diagnostic criteria, we speak of ‘early disease modification’ in this group rather than disease prevention. Even so, delaying the onset of overt PD, if only by a few years, would be a major achievement with clear clinical benefits. Evidently, the ideal timing of the trial also depends on the intervention target and the specific target population. PD is a heterogeneous disease, in which treatment benefits may differ per patient sub-group depending on risk profile. Hence, the current movement towards more personalized PD trials [2].

Second, it is also possible to define an earlier at-risk population that consists of asymptomatic individuals who are at increased risk to develop PD in a more remote future, the advantage being that putative disease-modifying treatments would theoretically have a greater effect here. Such prediction models would typically include genetic markers and midlife environmental risk factors, such as sex and non-use of caffeine. However, the accuracy of such models to predict clinical PD at the level of an individual is currently poor, especially within a limited timeframe [20, 21]. Some of the early disease modification AD trials, therefore, focused on recruiting only those with a autosomal dominant genetic predisposition for the disease [22]. However, for both AD and PD, autosomal dominant genetic predispositions are very rare—especially when narrowing it down to specific subtypes [23]. Such a focused recruitment strategy is thus challenging from a practical perspective and the trial results will not be representative for the vast majority of patients.

Third, combining clinical features and risk factors in trial recruitment criteria is currently considered the ideal option, being a compromise between predictive power and adequate room for prevention purposes, as reflected by the International Parkinson and Movement Disorder Society (MDS) research criteria for prodromal PD [3]. It should be noted that many risk factors for PD are also associated with Lewy Body dementia and multiple system atrophy, which increases the chance that participants with early symptoms are misdiagnosed as ‘prodromal PD’ in the recruitment process. This would negatively affect the reliability of clinical trial sample size calculations. To avoid a high screen failure in recruitment for these trials, previously set-up recruitment registries might be used to selectively invite those known to have certain PD-risk factors, although the true value of such registries as a basis for recruitment is yet to be shown [24].

Finally, for immunotherapy trials targeting α-synuclein in particular, ideally, only individuals with abnormal levels of α-synuclein would be recruited, because the intervention may only be effective in this population. However, the diagnostic accuracy of measures of elevated α-synuclein levels in CSF for PD pathology remains contentious [25, 26]. Positron emission tomography (PET) scans and serum and plasma biomarkers to measure α-synuclein levels are currently being developed, e.g., the real-time quaking-induced conversion (RT-QuIC) technique, but are not available for use in trial settings yet [27]. For serum and plasma biomarkers, the dynamics in fluid measures are unknown [28], but PD biomarker study results will be announced soon [29]. Given the many exceptions of α-synucleinopathy staging, according to the Braak hypothesis, it cannot be used as a tool to predict future symptoms [30]. Misdiagnosis in the recruitment process should be avoided to lower the noise in clinical trial power calculations, and thereby improve the chance of finding the potential effect of an intervention and limit the minimal number of participants.

### What to tell research participants?

According to current research criteria, someone has ‘prodromal’ PD when summing up the presence of (clinical) risk factors, such as olfactory loss and non-use of caffeine, indicates that someone’s overall risk to develop the disease is exceptionally high, up to a presumed risk > 80% if many risk factors are present [3, 5]. Since the word ‘prodromal’ refers to an early stage of a disease, i.e., before full manifestation of symptoms, the interpretation is that being at high risk for PD is the same as being in an early stage of the disease. Consequently, high-risk individuals recruited for trials can be labeled as having an early or ‘prodromal’ stage of a brain disease, while they would not have received such a diagnosis in a clinical setting—similar to the current situation in AD prevention trials.

We learned from the AD field that this scenario is vulnerable to miscommunication. Despite elaborate education sessions, some cognitively healthy research participants did not understand that having biomarkers of amyloid-β indicates an increased but uncertain risk for dementia [31]. Moreover, telling people they are in an early stage of a neurodegenerative disease, rather than being at increased risk, may have consequences for how they understand themselves and how they are perceived by others [32]. Some people may start thinking of themselves as “sick” and feel less able to take part in certain activities, for example, or colleagues will perceive them in that way, even if such an early diagnosis reflects, in fact, risk status. Since a diagnosis of a neurodegenerative disease may also provide a reason to “excuse” someone from societal or work obligations, expanding the disease label may create new challenges for employment and insurance policies, as well. If clinical practice will gradually adopt the expansion of PD diagnostic criteria applied in research, as occurred in the AD field, this may have severe social and psychological disadvantages [15, 33]. Moreover, substantial financial investments will then be needed for diagnostic tools and, potentially, further monitoring and follow-up [28]. We, therefore, propose to stick to terms of ‘risk’ rather than ‘prodromal’ (or ‘preclinical’) disease, also to prevent discrepancies between research and clinical practice.

For some, being informed of being at increased risk for a neurodegenerative disease may cause anxiety and stress [34]. For others, in the case of PD rather than AD, it could offer relief by providing an explanation for the psychological or physical difficulties that people experience in the years before receiving a diagnosis [35, 36]. Whether an early diagnosis is preferable, i.e., timely, will depend on the individual [37]. In the absence of an effective treatment, however, the majority of patients with PD are sceptical about early risk disclosure [38]. People who received an increased AD risk status contemplated a change to their health behavior, living situation, or future plans more compared to those who did not, even though the validity of this risk status for future symptom development is still uncertain [39].

Future trials including those at risk for PD can benefit from thought-out communication strategies from the AD research field to inform research participants of their risk status [40, 41], and should incorporate research on the impact of knowing to be at risk in the case of PD. A key challenge in communicating risks with patients is to adequately convey the limited diagnostic accuracy of currently available biomarkers and the precariousness of risk algorithms. Furthermore, participants should have legal safeguards against discrimination based on their PD-risk status and to protect their privacy. These are among the reasons why risk algorithms, for AD as well as PD, may be less suited for clinical practice, while being useful for recruitment purposes of upcoming clinical trials.

## How promising are immunotherapy PD trials targeting α-synuclein?

Before exposing relatively healthy people to the risks and burdens of PD prevention or early disease modification research, particularly in the case of immunotherapy, and before investing substantial resources, it should be reasonably plausible that the intervention under study will lead to a health benefit. How strong is the pre-trial evidence of immunotherapeutic agents currently selected for PD prevention trials in people with no or only mild complaints? And how does that compare to the presumed high plausibility of previously tested anti-amyloid-β interventions in AD research, which so far have failed to lead to a tangible health benefit?

The most promising target for early intervention is α-synuclein aggregation in the brain, which is strongly associated with most motor symptoms of PD [42, 43]. A causal role for α-synuclein aggregation in the disease process of PD seems highly plausible, since genetic variants strongly associated with PD determine α-synuclein levels and folding [44]. Moreover, in a mouse model of PD, anti-α-synuclein immunotherapy which reduced α-synuclein aggregation also reduced neurodegeneration [45]. For prevention trials specifically, α-synuclein seems a suitable intervention target, because it plays a crucial role in the stages of PD prior to neurodegeneration [9]. On the other hand, clinical disease severity in PD is not directly linked to reduced α-synuclein levels in CSF [46] and, similar to amyloid-β in AD, aggregation of α-synuclein may also be an epiphenomenon rather than the pathophysiological cause of neurodegeneration [44].

The safety and tolerability of anti-α-synuclein immunotherapy have been established in humans [47]. Recently, the PASADENA study results showed that the anti-α-synuclein antibody Prasinezumab (RO7046015/PRX002) did not lesson symptom worsening after 1 year in participants with early PD (NCT03100149) [48]. Other immunotherapy phase 2 trial results in recently diagnosed PD patients are underway (SPARK Study, ClinicalTrials.gov identifier NCT03318523). Even if these phase 2 trials suggest beneficial clinical effects, expectation management will be vital. Successful removal of a presumed causally related protein does not necessarily lead to improved functioning, as we have seen in AD trials. Similarly, highly promising phase 2 trial results may not result in clinically detectable effects in phase 3 trials [49].

Previous AD immunotherapy trials have been criticized for their strong reliance on amyloid-β to define and diagnose AD [50], especially after their results showed that the removal of amyloid-β had no positive clinical effect [51]. In light of the disappointing results of these trials, it seems wise for upcoming PD immunotherapy trials not to rely too heavily on the role of α-synuclein alone in causing the symptoms of PD, and continue to focus on co-investigating multiple other intervention targets. A key argument for the latter is the strong inter-individual variation between biomarker levels and PD symptoms [30], which increases the likelihood that other biological processes are being involved.

Another likely contributor to PD is dysregulation of the immune system, in which α-synuclein may also play a central role. Possibly, the immune response is triggered in PD by pathogenic forms of α-synuclein [52]. In AD, anti-amyloid-β treatment led to sometimes severe inflammatory responses in the brain, ranging from MRI changes without symptoms to fulminant fatal encephalitis. Whether such an inflammatory response may occur following anti-α-synuclein treatment is uncertain, but it has been suggested that these side-effects might be avoided in future PD trials with the right participant selection [53].

Infectious triggers in the gut microbiome are another, more recent focus in the search for potential intervention options linked to the immune system, given its link with brain inflammation in PD [54, 55]. How these links with PD may translate into a potential intervention for PD is, however, still uncertain. Rather than aiming to modify the biological root of PD, future interventions may also aim to foster functioning neurons and protect them from the damaging effects of α-synuclein [54]. Interventions following this strategy can be sought in repurposed drugs [56, 57], such as Exenatide [58]. Given a strong genetic link between LRRK2 and PD, LRRK2 kinase inhibitors are also investigated as a potential treatment. Phase I study results showed that a LRRK2 inhibitor can substantially lower kinase activity, which might be neuroprotective [23]. Challenges for this treatment strategy include the lack of measures for LRRK2 activity so far, and the potential for adverse peripheral side-effects [23]. Potential biomarkers for target engagement are currently being explored, which may also lead to the selection of patient subgroups that may have a greater benefit from treatment [2]. Ambroxol, a repurposed drug known for respiratory disease treatment, may become an important therapy for those with a mutation on the glucocerebrosidase gene (GBA) that increases one’s risk to develop PD [59]. However, only the small minority of PD patients who carry this genetic risk factor may benefit from this therapy and phase III trial results still have to measure Ambroxol’s impact on PD motor features.

## Trial design

### Recruitment and retention strategies

AD prevention trials have encountered major logistical challenges due to slow recruitment, high screen failure rates, and low retention. Immunotherapy trials that selected only those with elevated levels of amyloid-β have taken 3 years to complete enrollment, with screen failure rates up to 90% [60], partly due to the exclusion of persons with co-morbidities [61]. Setting up large transnational recruitment registries with clinical and biomarker information of potential research participants could be set up to tackle these issues [62]. However, the success of such approaches remains to be determined in the AD field, and it poses new ethical challenges if a small minority of those who registered for these trial-readiness cohorts will ever be eligible for a clinical trial. Moreover, designing such a registry and linking personal data on risks for future PD development requires careful consideration of ethical issues related to privacy, informed consent, and disease risk disclosure [63]. When informing people that they have an increased risk for a progressive and debilitating neurodegenerative condition and then invite them to participate in a trial of an agent that might mitigate that risk, people might feel more inclined to take part. Researchers should be warranted in these cases for false hope on the side of the potential participant.

### Outcome measure selection

For the upcoming trials that intend to investigate anti-α-synuclein immunotherapy in individuals with ‘prodromal’ PD [16], there is already some evidence for the short-term safety and tolerability for certain anti-α-synuclein antibodies [17, 47]. However, it remains essential to consider safety outcomes in the upcoming phase 1 and phase 2 trials. In AD trials, side-effects of immunotherapy included very serious adverse events, including meningoencephalitis and brain microhemorrhage [64]. Some were not detected until the phase 3 trial [65]. Future immunotherapy PD trials should, therefore, anticipate on these and other serious side-effects, and continue to monitor potential imaging abnormalities during phase 3 trials with the scrutiny level of a phase 1 or 2 trial.

The healthier the target population and the further away from a diagnosis of PD, the more difficult it is and the longer it will take to establish a clinical effect of the intervention. To have sufficient power to detect a clinical effect, there are two main possibilities: first, to design trials with a duration as long as 5–10 years, which carries a high risk of drop-outs and for which it is difficult to attract funding; second, to enroll a very high number of subjects, which could limit the trial duration to a shorter period, but at exponentially high costs. Modeling long-term effects based on small effect sizes for clinical or even biological outcomes in short-duration studies remains treacherous and may result in overestimating potential treatment effects, especially when outcome measures may leave room for a certain placebo effect [66].

The movement disorder society—unified Parkinson's disease rating scale (MDS-UPDRS) has long been the most common clinical outcome measure in PD clinical trials, but its scoring comes with several methodological challenges [67]. Importantly, this scale cannot detect changes in the prodromal stages of the disease due to floor effects.

Alternatively, documenting an effect on a biomarker targeted by the intervention, as measured either in body fluid or using neuroimaging as a proxy outcome measure, seems attractive, and has been applied. However, similarly to biomarkers of AD, PD biomarkers are not directly related to clinical parameters and are, therefore, no reliable indicator of clinical efficacy [26, 30, 46]. This can partly be explained by the fact that it remains a fundamental question whether PD’s range of clinical symptoms results from one common, or many pathophysiological processes [68, 69]. If the latter becomes more likely, PD research will presumably become increasingly individualized [57].

More sensitive outcome measures, e.g., changes in motor performance as detected with modern technologies including wearables, could increase the possibility to detect small potentially relevant effects of an intervention [70]. Still, any effect on an intermediate outcome which is not directly translatable into clinical relevance can at best only serve as proof of concept. Intermediate biomarker or digital monitoring outcomes will, therefore, not suffice in phase 2 trials when targeting the early disease stages of PD, i.e., with subtle clinical features, where an indication of a clinical effect at the level of relevant symptoms remains desirable [65].

Finally, similar to AD trials, before any drug is approved, an effect on a patient-oriented clinically meaningful endpoint is highly desirable, such as an effect on activities of daily living (ADL) or quality of life (QOL). This is challenging in its own right. For example, a recent trial of exercise—another intervention with potentially a disease-modifying and preventive potential—showed the stabilization of MDS-UPDRS motor scores in patients with the early stage PD, with an effect size that exceeded the minimal clinically important difference, yet without concurrent improvements in quality of life [71]. QOL outcome measures could be combined with other physical and psychological symptoms and life impact measures to better reflect the disease state [72].

As it would be difficult to establish a clinical consequence in a preclinical population, the Food and Drug Administration (FDA) is exploring alternative outcome measures for accelerated approval in the case of AD prevention trials [73]. These discussions may open the path for approval based on more subtle outcome measures in PD trials, although it also carries the risk of descending into the approval of therapies which have never shown to be effective in terms of patient benefit.

### Advancing to phase 3 trials

In AD research, anti-amyloid-β immunotherapy trials showed no improvement of cognition or functional ability in phase 2 after proof of concept was established. Even so, based on post hoc sub-group analyses, the tested antibodies advanced to phase 3 trials, which have all led to disappointing results [12]. Looking back, the misleading expectations resulted from an over-interpretation of subgroup analyses [65]. In light of these occurrences, the PD field should be warned against spending scarce resources on phase 3 trials in the absence of strong suggestions of clinical efficacy in phase 2 trials, and apply the appropriate sub-group analyses guidelines to avoid the over-interpretation of results.

## Conclusion

Similar to the movement in AD research, PD research is now focussing on an earlier (even prodromal) diagnosis in the hope that intervening in an early stage may slow down or possibly even arrest the disease process in those with no or only mild symptoms. This strategy shift is accompanied by new challenges that have hampered progress in the field of AD in recent years, where a similar research strategy led to a series of disappointing trial results. In this paper, we have provided guidance on how we can capitalize on lessons and experiences from AD research in the field of PD, such as how to inform people of their risk status and how to deal with the ethical challenges of trial-readiness cohorts. We also draw attention to the possible impact that PD-risk algorithms—developed with good intentions for research purposes—may have on persons in clinical practice. Taken together, we anticipate that consideration and implementation of these lessons and experiences will accelerate progress for people at risk of or living with PD.
